# Spatial and temporal distribution of American cutaneous leishmaniasis in Acre state, Brazil

**DOI:** 10.1186/s40249-017-0311-5

**Published:** 2017-06-07

**Authors:** Leonardo Augusto Kohara Melchior, Andréia Fernandes Brilhante, Francisco Chiaravalloti-Neto

**Affiliations:** 1 Biological and Nature Science Center, Federal University of Acre, Rio Branco, AC Brazil; 2 School of Public Health, University of São Paulo, São Paulo, SP Brazil; 3 Department of Epidemiology, School of Public Health, University of São Paulo, São Paulo, SP Brazil; 4 Departamento de Epidemiologia, Faculdade de Saúde Pública, Universidade de São Paulo, Av. Dr. Arnaldo, 715, CEP 01246-904, São Paulo, SP Brazil

**Keywords:** Cutaneous leishmaniasis, Geographical information system, Spatial analysis, Scan statistics, Acre, Brazil

## Abstract

**Background:**

Acre has reported the highest incidence of American cutaneous leishmaniasis (ACL) in Brazil in recent years. The present study seeks to identify high and low risk agglomerations of ACL in space and space-time during the period from 2007 to 2013 in Acre, and also to characterize the occurrence of the disease in time and according to sociodemographic variables.

**Methods:**

This is an ecological study, the study population of which consisted of autochthonous ACL cases notified in the municipalities of Acre by an epidemiological surveillance system. Scan statistics of SaTScan™ software were used to identify spatial and space-time clusters. In addition, the cases were characterized by sex, age, home situation (in a rural or urban area), and temporal tendency.

**Results:**

Acre reported an incidence rate of 12.4 cases per 10 000 inhabitant-years in the study period, with the rates varied greatly (standard deviation of 21.8) among their 22 municipalities. One agglomeration of high risk and three of low risk were detected in space and space-time. Four of the five micro-regions of Acre presented a stationary temporal tendency. The profile of transmission varied according to the micro-region. Generally speaking, the disease occurred more often among young people, those of male gender, and those living in rural areas.

**Conclusions:**

Acre has stood out within the Brazilian national context due to its high rates of ACL incidence in the central region of the Acre Valley. The high rates in the micro-region of Brasiléia are related to the disease’s intra/peridomiciliary occurrence, and it would seem that the municipality of Sena Madureira is approaching a transmission pattern similar to that of Brasiléia. In other micro-regions, the profile of the disease’s transmission is mainly related to the forest/sylvatic cycle of ACL.

**Electronic supplementary material:**

The online version of this article (doi:10.1186/s40249-017-0311-5) contains supplementary material, which is available to authorized users.

## Multilingual abstracts

Please see Additional file 1 for translations of the abstract into six official working language of the United Nations.

## Background

American cutaneous leishmaniasis (ACL) is a metaxenic disease widespread throughout the world. It is caused by the infection of various species of protozoa of the genus *Leishmania*. Considered to be a neglected tropical disease, ACL is transmitted to humans through the bite of infected female phlebotomine sandflies [1]. Brazil is one of 10 countries (alongside Afghanistan, Algeria, Colombia, Iran, Syria, Ethiopia, Northern Sudan, Costa Rica, and Peru) that has the greatest numbers of cases notified, accounting altogether for 70–75% of the global incidence of the disease [1].

In Brazil, between 2007 and 2013, the disease was verified in practically all states, the largest number of new cases being registered in the states of Bahia (22 255), Pará (19 930), and Mato Grosso (15 144). However, the state of Acre, with 5 689 new cases notified in that period, accounted for the highest rate of incidence (11.1 cases/10 000 inhabitant-years); this rate was 1.6 times that of Mato Grosso (7.1 cases/10 000 inhabitant-years), 2.6 times the incidence of Legal Amazonia (4.3/10 000 inhabitant-years), and 12.1 times the incidence in the whole of Brazil (0.9/10 000 inhabitants-years). Acre has reported the highest rates of ACL incidence in the country since 2001 [2].

According to the American Cutaneous Leishmaniasis Surveillance Program, in Brazil, one of the specific objectives of surveillance is to identify and monitor territorial units of epidemiological significance [3]. Therefore, geographical information systems (GISs) and spatial analysis, in view of their usefulness in terms of understanding and visualizing the epidemiological behaviour of diseases such as leishmaniasis, have been important tools in achieving these objectives both in Brazil [4–6] and in other countries [7–10].

The present study seeks to identify high and low risk agglomerations of ACL in space and space-time in the period from 2007 to 2013 in the municipalities of Acre, and also to characterize the occurrence of the disease in time and according to sociodemographic variables.

## Methods

### Study area

Situated in the Amazon region, Acre has a surface area of 164 123.739 km^2^, corresponding to 1.92% of Brazil’s national territory. Historically, the state’s economy has been based on vegetable extraction, especially in the export of rubber and chestnuts, which has contributed to the implantation of numerous settlements in the region. Agriculture and fishing are also important.

The state is situated on a plateau with an average altitude of 200 m above sea level. Its native vegetation is tropical forest and it has a hot, humid equatorial climate. The average annual temperature is 31.5 °C and the total annual rainfall is 2 100 mm [11].

**Fig. 1:**
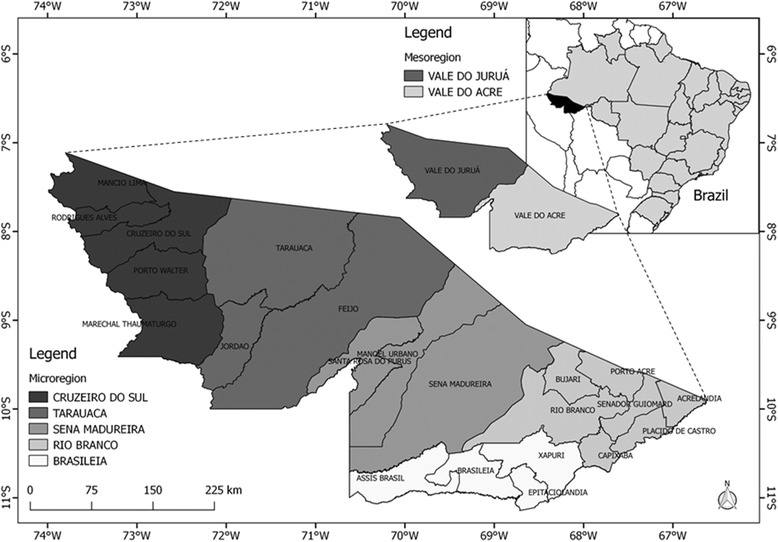
Map of the study area of Acre and its municipalities and territorial divisions in meso- and micro-regions

The state is divided according to the Brazilian Institute of Geography and Statistics (Instituto Brasileiro de Geografia e Estatística, IBGE) into two large meso-regions, which are subdivided into five micro-regions [12]. The micro-regions of Rio Branco, Sena Madureira, and Brasiléia constitute the meso-region of the Acre Valley, while the micro-regions of Cruzeiro do Sul and Tarauacá constitute the meso-region of the Juruá Valley (see Fig. 1). In 2016, the state had 816 687 inhabitants residing in 22 municipalities [12].

### Data collection

This ecological study was based on data extracted from the Information System of Notifiable Diseases (Sistema de Informação de Agravos de Notificação) and that of the Health Secretariat of the State of Acre (Secretaria de Estado de Saúde do Acre). The study population consisted of autochthonous ACL cases who resided in the municipalities of Acre from 2007 to 2013. To better understand the epidemiology of ACL in the state, the distribution of cases by sex, age, and home situation (in a rural or urban area) was also studied. Information on the number of inhabitants, and their ages and sex, from all municipalities, was extracted from the IBGE.

### Spatial and epidemiological analysis

To identify spatial and space-time clusters, we used scan statistics by means of SaTScan™ version 9.4.2 software [13]. The analytical technique of this program permits one to test whether one should accept the alternative hypothesis (H_A_ = individuals of a particular area are more likely to be affected by the disease) or to maintain the null hypothesis (H_0_ = all individuals of a given population are equally likely to be affected by the disease) [14].

Three data banks were connected to the program for the analysis: one with the latitudes and longitudes of the centroids of each municipality, another with the populations of the municipalities by year, and a third with the number of cases per municipality per year [14].

For the identification of the spatial agglomerations, we used the Kulldorf’s scan statistic [14] and considered a Poisson probability distribution, with the following settings: non-occurrence of geographical overlapping of clusters, maximum spatial size of each cluster equal to 50% of the exposed population, circular clusters, and a Monte Carlo procedure with 999 repetitions to obtain *P*-values. This model only takes into consideration the space in which the cases occurred [14].

We also used the SaTScan^TM^ program to obtain space-time clusters. For this, we used the same conditions and settings as for the spatial clustering with two more conditions: maximum temporal size of each clusters equal to 50% of the study period and the measurement of time standardized in years [15].

Both the space scanning and space-time techniques were adjusted according to the population of the municipalities and set to detect agglomerations of high and low risk for ACL. The test of significance of the agglomerations identified was based on the comparison of a null distribution obtained by the Monte Carlo simulation and the statistical ratio test of verisimilitude [16]. In order to compare different areas with each other, the program calculated the relative risk (RR) of each agglomeration, representing the ratio of the ACL incidence inside the cluster and the incidence outside the cluster. The agglomerations of high and low risk were considered statistically significant at the level of 5% and were presented asthematic maps developed with the free software QGIS 2.12.3 ‘Lyon’ based on the maps made available by the IBGE.

Temporal analyses were undertaken on the basis of the number of cases to estimate the tendency of the disease (whether increasing, stationary, or diminishing) by municipality, micro-region, meso-region, and in the state as a whole during the study period. The relatively short period of seven years (from 2007 to 2013) only permitted one tendency to be calculated for each locality. The analyses were carried out with the Stata 13 statistical program using the autoregressive method of analysis known as Prais-Winsten. This procedure corrects the temporal auto-correlation of the first order of the residuals. The result of this analysis is the annual percentage alteration called the rate of annual increment and its respective 95% confidence interval. The tendency is considered decreasing if both values of the confidence interval are negative, increasing if these values are positive, and stationary when the confidence interval contains the zero value [17].

The other variables analysed were: municipality, year, domestic situation (in a rural or urban area), sex, age, schooling, type of entry (new cases, relapses, etc.), clinical form, coinfection with HIV, criterion (laboratorial or clinical), and methods of diagnosis and progress of the disease. The organization and analysis of the data were undertaken using Microsoft Excel 2016 and the descriptive statistical analysis of the variables was conducted using Stata 13 [18].

## Results

Acre reported an incidence rate of ACL of 12.4 cases per 10 000 inhabitant-years in the study period. These rates varied greatly (standard deviation of 21.8) among the 22 municipalities of Acre and ranged from a minimum of 3.1 in Cruzeiro do Sul to a maximum of 89.1 in Assis Brasil (see Table 1).

**Table 1:** Distribution of ACL cases by incidence, number of cases and temporal tendency in the meso-regions, micro-regions, municipalities, and the state of Acre as a whole, 2007 – 2013

Locality	ri	*n*	%	ARI	95% *CI*	Tendency
State						
Acre	12.4	6 257	100	-1.2	-1.9; -0.5	Diminishing
Meso-region						
Acre Valley	13.6	4 904	78	-1.8	-2.7; -0.9	Diminishing
Juruá Valley	9.4	1 353	22	+0.2	-2.9; +3.3	Stationary
Micro-region and municipalities						
***Brasiléia***	***45.5***	***1 837***	***29***	**-** ***0.8***	**-** ***1.6; +0.0***	***Stationary***
Assis Brasil	89.1	371	6	-2.8	-4.6; -1.1	Diminishing
Xapuri	81.9	903	14	-0.8	-1.2; -0.4	Diminishing
Brasiléia	29.4	434	7	+0.7	-2.4; +3.9	Stationary
Epitaciolândia	12.4	129	2	+1.7	-0.7; +4.0	Stationary
***Sena Madureira***	***29.6***	***1 036***	17	***+1.8***	**-** ***0.3; +3.9***	***Stationary***
Manoel Urbano	29.8	163	3	-3.0	-5.6; -0.4	Diminishing
Santa Rosa do Purus	18.5	60	1	-2.2	-16.6; +12.3	Stationary
Sena Madureira	31.0	813	13	+3.3	+1.5; +5.1	Increasing
***Tarauacá***	***13.2***	***684***	11	**-** ***1.5***	**-** ***3.0; +0.1***	***Stationary***
Feijó	16.8	380	6	-2.6	-7.6; +2.4	Stationary
Jordão	19.3	89	1	-2.9	-10.1; +4.3	Stationary
Tarauacá	8.8	215	3	-4.1	-15.8; +7.6	Stationary
***Cruzeiro do Sul***	***7.3***	***669***	11	***+2.7***	**-** ***2.1; +7.5***	***Stationary***
Cruzeiro do Sul	3.1	169	3	+7.8	-1.0; +16.5	Stationary
Mâncio Lima	17.8	189	3	+6.6	+4.3; +9.0	Increasing
Marechal Thaumaturgo	21.9	221	4	-2.7	-7.9; +2.5	Stationary
Porto Walter	4.8	31	0	-19.4	-29.4; -9.4	Diminishing
Rodrigues Alves	5.9	59	1	+7.1	-3.8; +17.9	Stationary
***Rio Branco***	***7.1***	***2 031***	32	**-4.5**	**-5.8; -3.2**	***Diminishing***
Acrelândia	12.4	108	2	-5.6	-12.0; +0.7	Stationary
Bujari	30.8	169	3	-5.9	-6.3; -5.5	Diminishing
Capixaba	24.0	153	2	-1.9	-14.3; +10.6	Stationary
Plácido de Castro	9.7	120	2	-7.2	-16.6; +2.3	Stationary
Porto Acre	14.5	151	2	-11.2	-14.8; -7.7	Diminishing
Rio Branco	5.5	1 264	20	-3.3	-5.6; -1.1	Diminishing
Senador Guiomard	4.7	66	1	-1.9	-11.3; +7.5	Stationary

ri = rate of incidence (cases/10 000 inhabitants/year); *n* = number of cases in the period; (%) = state contribution (percentage); ARI = annual rate of increase (percentage);95% *CI* = confidence interval of 95% (percentage); Tendency = interpretation of the tendency; the names of the micro-regions and their results are in bold and italic

The meso-region of the Acre Valley reported the highest rate of incidence, as a result of the high incidences reported in the micro-regions of Brasiléia and Sena Madureira (see Additional file 2: Table and Additional file 3: Graph). The municipality of Xapuri stood out in terms of incidence due to the number of cases, however, the municipality of Assis Brasil reported the highest ACL incidence in the period. The municipality of Rio Branco reported the largest number of cases, followed by Xapuri, Sena Madureira, Brasiléia, Feijó, and Assis Brasil. These six municipalities accounted for 67% of all cases reported (see Table 1).

**Fig. 2:**
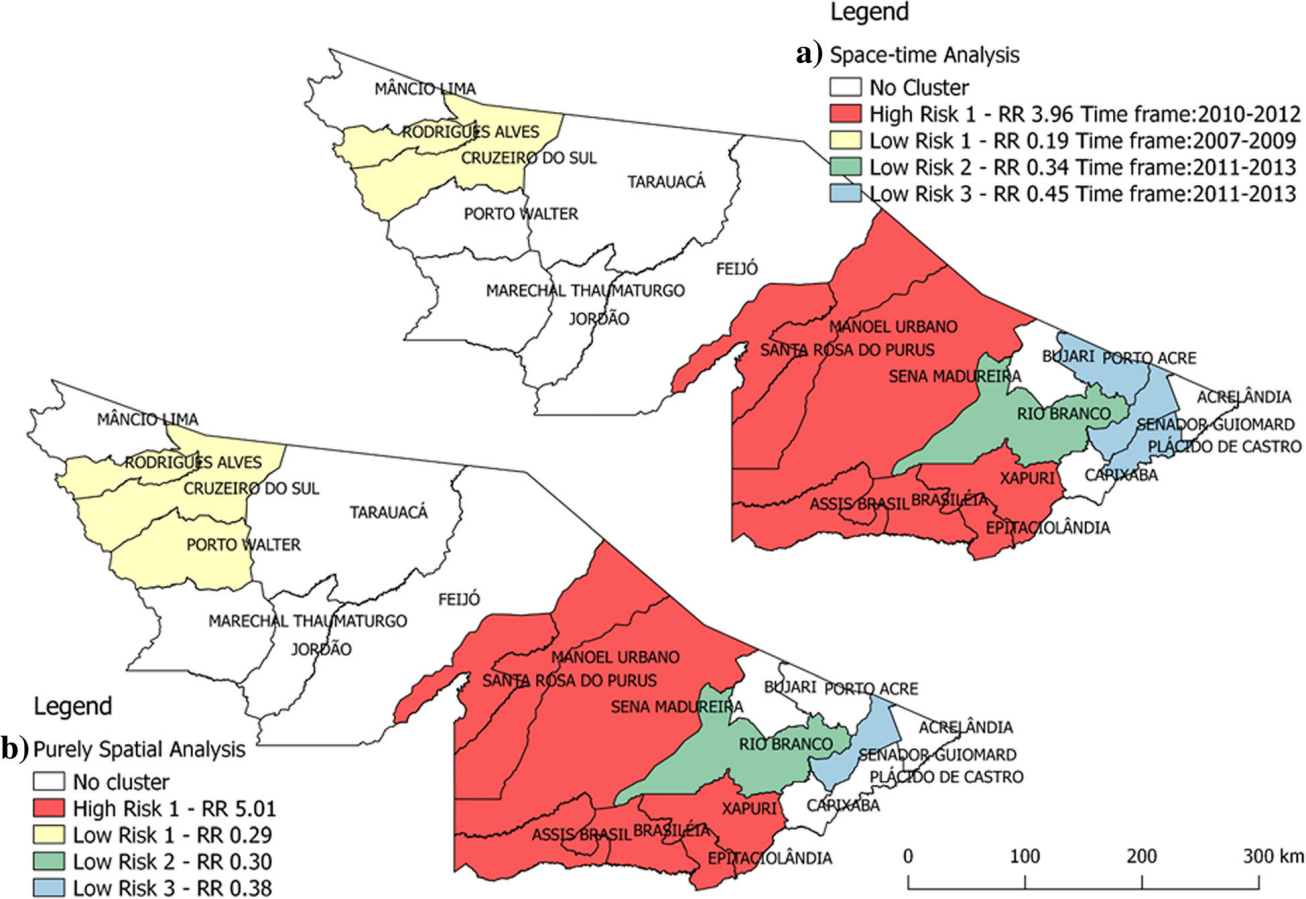
Locations of the detected clusters of ACL cases, based on the a) space-time analysis and b) purely spatial analysis, Acre state, 2007 – 2013

During the period investigated, agglomerations of high risk in space and in space-time were identified (2010–2012). These clusters encompassed the micro-regions of Brasiléia and Sena Madureira (see Fig. 2 and Additional file 4: Figure). Further, three low-risk clusters were found, both in space and space-time, two of them located in the micro-region of Rio Branco and the other composed of municipalities of the micro-region of Cruzeiro do Sul.

It is noteworthy that both maps in Fig. 2 are very similar, but they have an important difference: the low risk spatial cluster with RR equal to 0.38 (cluster number 3 in Fig. 2b) was composed of only one municipality (and worked during the whole study period), but the low risk spatiotemporal cluster with RR equal to 0.45 (cluster number 3 in Fig. 2a) was composed of three municipalities (and worked from 2011 to 2013).

In accordance with the temporal analysis of the rates of incidence, Acre presented a decreasing tendency in the number of ACL cases in the period from 2007 to 2013, as also did the Acre Valley and the micro-region of Rio Branco. The municipalities of Rio Branco, Xapuri, Assis Brasil, Manoel Urbano, Bujari, Porto Walter, and Porto Acre contributed to the state’s decreasing tendency, while the municipalities of Sena Madureira and Mâncio Lima reported an increase in the number of cases. The other localities presented a stationary tendency regarding the number of cases (see Table 1).

Analysing the incidences in terms of the domiciliary situation, it can be seen that the residents of rural areas were most affected in almost all micro-regions: Brasiléia (83.2%), Cruzeiro do Sul (71.3%), Tarauacá (70.9%), and Sena Madureira (70.1%). The only exception is the micro-region of Rio Branco (50.0%).

**Fig. 3:**
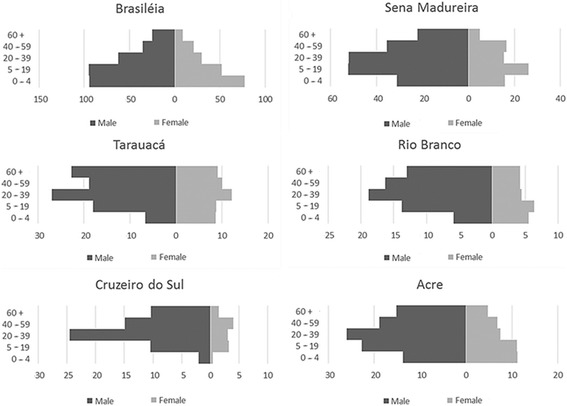
Distribution of ACL incidence in the micro-regions of Acre by sex and age group, 2007 – 2013

For a better understanding of how the disease affects each micro-region individually, the incidences were calculated by sex and age group, and are presented in Fig. 3. Regarding sex, a predominance of persons of the male sex was observed in all the micro-regions. In the state as a whole, the RR was 1.4 times greater for persons of the male sex than for those of the female sex. This risk remained stable throughout the period studied. The RRs for the sexes differed between the micro-regions: Brasiléia (1.7), Sena Madureira (2.1), Tarauacá (2.2), Rio Branco (2.8), and Cruzeiro do Sul (5.8).

Regarding the age group, it was observed that the Brasiléia micro-region formed a pyramid, whereby the incidence diminished as age increased for both sexes. In the micro-regions of Cruzeiro do Sul, Tarauacá, and Rio Branco, it formed a pattern in which the group with the highest was the 20 – 39 years old rather than at the extremities, thus forming a lozenge shape. However, among females, lesser homogeneity was observed between the age groups than among males. The micro-region of Sena Madureira presented an intermediate pattern, with the highest incidences occurring in the age group of 5 – 19 years for both sexes.

The high incidence among young people, mainly in the age group of 5 – 19 years in the two regions with the highest incidence (Brasiléia and Sena Madureira), affected the distribution of ACL incidence in Acre making this the second age group most affected by the disease in the state. The higher age groups (40 – 59 years and 60 years and above) were the most relevant in the micro-regions with the lowest incidences: Tarauacá, Rio Branco, and Cruzeiro do Sul.

The average age of ACL cases was 23.7 years, and the standard deviation was 16.8 years. About 10% of the cases were registered among children up to 4 years of age, 25% in children up to 11 years of age, 50% in persons up to 20 years of age, 75% in persons up to 33 years of age, and 90% in persons up to 48 years of age. Generally speaking, the population of the state had a low education level (59.2% of the school-age cases had incomplete basic schooling).

During the study period, the state reported 91% of new cases, 8% of relapses, and 1% of transfers or unknown. About 81% of the cases were of the cutaneous form of the disease, 18% of the mucosal form, and 1% of the mucocutaneous form. The occurrence of the mucosal form stood out in the Tarauacá micro-region (28%), which also reported one of the highest incidence coefficients, as compared with the others. For 91% of the cases, the criterion used for confirmation was the laboratorial one — direct parasitological (57%), Montenegro skin test (53%), and histopathological diagnosis (16%). In less than 1% of the cases, there was coinfection with HIV. About 96% of cases were declared to have been cured of ACL, 3% abandoned their treatment, and 1% followed other courses (death by other causes, transfers, and change of diagnosis). Sena Madureira reported the greatest number of dropouts from treatment, at about 10%. In the other micro-regions, this figure did not exceed 2.5%. The only death due to ACL notified occurred in the Rio Branco micro-region in 2007.

## Discussion

This study revealed important details about ACL in Brazil, namely that the state of Acre is an endemic zone of concern within the national context. In addition, the study showed that the incidence rates of the state’s municipalities are greatly varied and also revealed that the disease shows different patterns of occurrence. It is probable that the fragments of forest covering the state contribute to the maintenance of the disease and its high incidence rates in some localities, especially among the populations who are rubber extractors, riverside dwellers, fishermen, etc., whose survival depends on extractive and small-scale agricultural activities [19]. These populations leave very close to the forest so that they are susceptible to participate of the transmission cycle of ACL that occurs inside and in the border of the forest, i.e., the forest/sylvatic cycle of ACL.

The disease is spatially heterogeneously distributed throughout the state with areas of greater or lesser risk both in space and in space-time. The rates of incidence in the areas that compose the high-risk agglomeration (Brasiléia and Sena Madureira) were high and may be responsible for the leading position of Acre in the epidemiological ACL scene in Brazil. It is noteworthy that in Brasiléia and Sena Madureira, most of the 970 570 hectares of the Chico Mendes reserve is found. This reserve is intended for self-sustainable exploitation and conservation of exhaustible natural resources by resident populations, who are found to be the most affected by ACL in the state [20, 21].

The spatial high-risk agglomerates detected by scan statistics deserve special attention on the part of the municipal and state surveillance systems in their fight against this zoonosis, especially in the municipalities of Xapuri [20, 21], Assis Brasil [22], and Sena Madureira. It is important to highlight that the purely spatial cluster says nothing about any changes in the behaviour of the disease (the identified spatial cluster may be the same for a long period of time), but could be used to intensify control measures that, if applied in a high-risk cluster, could change the future behaviour of the disease [13, 15].

On the other hand, the spatiotemporal analysis is looking for high or low RRs in the space considering the temporal unit of analysis, i.e., a year or a sequential set of years. This technique provides the identification of changes in space and time without the necessity of having to draw maps for each year [13, 15]. The geographical coincidence between the spatial and spatiotemporal high-risk clusters (that should not necessarily have occurred) reinforces the result found in the purely spatial analysis and the necessity of prioritizing the surveillance and control measures of ACL in these localities [20–22].

Despite the capital, Rio Branco, reporting the largest number of ACL cases in the state due to its large population, it was classified as low risk area for the occurrence of ACL in the analysis of spatial and temporal agglomerations. However, it is likely that there are places within the Rio Branco micro-region where transmission is intense and it would be necessary to carry out a specific spatial analysis to locate these high-risk clusters.

Although the state presents a diminishing temporal tendency, four of the micro-regions have been stationary during the last seven years studied. This seems that the strategies adopted for the control of ACL are not being efficient in reducing the number of cases. This is related to the great difficulties faced in attempts made to control the disease in the region in view of particular conditions of Amazonia, such as to control the phenomena in a forest area, the difficulty of accessing health services especially in the rainy season, and the proximity of dwellings of rural areas to forests [19].

In Cruzeiro do Sul, Tarauacá, and Rio Branco, the RR is much greater among men, especially those who are within the productive age group. This suggests that in these micro-regions, the exposure to the vector occurs mainly in manual labour activities and those men who do certain occupations are at greater risk of contracting the disease [21, 23]. Given the high rate of occurrence of ACL in rural areas in all micro-regions, it can be deduced that the disease is related to forestry and farming activities. The human interaction with the forest has been associated with a high risk of infection by *Leishmania*, known as the forest/sylvatic cycle of ACL [24]. This is the main profile of the disease transmission in these micro-regions.

Secondarily, in Cruzeiro do Sul, Tarauacá, and Rio Branco, an intra/peridomiciliary disease transmission pattern was observed. This may be seen by the endemic character of the disease in children, women, and the elderly. The greater homogeneity existing between the groups of females of all ages probably occurred because women are less exposed than men to activities undertaken in forests [24, 25]. However, it is reaffirmed there are two patterns of transmission in these micro-regions. The first is related to occupational activities related to forestry, engaged in mostly by men, and the second is related to intra/peridomiciliary transmission contributing to the high incidence mainly among children and women. These patterns have also been reported in other regions of Brazil [26, 27].

On the other hand, the high-risk cluster in Brasiléia was determined by transmission mainly in intra/peridomiciliary environments, a hypothesis supported by the fact that this micro-region has presented the lowest RR for males. In this region, the highest incidence was among residents of rural areas and the groups most affected were children and young people. This age group’s cellular and humoral immaturity may render it more susceptible to infections [28].

We also observed that age group of 40 years or above were less affected in some regions, especially in Brasiléia. Perhaps this can be explained by the more frequent exposure to constant bites of sandflies over the years, when contact with the saliva of this insect may provide some protection against clinical forms of the disease [29].

Due to the activities exercised by inhabitants of Brasiléia, mainly extracting chestnut and rubber, and farming activities, and also because their dwellings are situated very close to forests, the inhabitants of this micro-region have an increased probability to come into contact with wild reservoir animals of *Leishmania* and vector. Additionally, domestic animals such dogs and cats may act as secondary reservoirs of the etiological agents of ACL in their domiciliary and peridomiciliary environments where vectors may become infected by *Leishmania* spp. when biting these domestic animals [24]. Further, in this context, the chances of humans coming into contact with vectors may be amplified due to increases in their population density as a result of a great number of available food sources (represented by domestic and synanthropic animals). These chances also increase due to the fact these people live in the proximity of the forest, allowing insects to keep their breeding and adult resting sites in this environment where they are all adapted. This may all favour the establishment of the intra/peri-domiciliar transmission cycle of ACL [30–32].

Regarding the age group most affected in Sena Madureira (5 to 39 years old), it is probable that this micro-region is undergoing a transition to a situation similar to that which is occurring in Brasiléia, with exposure to the vector coming ever closer to the dwellings, i.e., in the intra/peri-domiciliary environment. The expansion of the extractivist and agricultural industries leads to an ever greater insertion of the traditional populations (chestnut and rubber extractors, farmers, etc.) into risk areas, thus leading to greater incidences of cases of the disease [33]. The municipality of Sena Madureira is the only component of the high-risk agglomerate that presents a growing tendency in the number of cases.

In terms of the state inhabitants’ socioeconomic level, it may be presumed that the majority of those who suffer from ACL are of low socioeconomic level, however, few studies deal in depth with the relationship between these conditions and zoonosis [34].

In comparison with the study undertaken in 2009 by Silva and Muniz [21], who assessed ACL cases in Acre in the period from 2001 to 2006, it may be noted that the proportions of cases confirmed by laboratory tests and of relapses were similar to those found in this study. The same occurred with cases who abandoned treatment, with the exception of Sena Madureira, where this proportion rose from 4.7 to 10.0%, between the two studies. A reduction in the number of cases of the clinical mucosal form was also observed, from 25 to 17%, as well as an apparent reduction in mortality, seeing that in the former study seven deaths occurred and in the present study just one did.

This study had some limitations related to the use of secondary data, which presents problems such as fields being either left blank or incompletely filled in on forms, information omitted or unknown, and possible under-notification. It is worth noting that the failure to undertake a differential diagnosis, useful for the confirmation of cases, may result in a super-notification of false cases, especially in regions recognized as being endemic.

Another limitation of the study was the use of the municipality as a spatial unit to evaluate the distribution of ACL cases. Due to the small number of units (the 22 municipalities of Acre), this approach could not capture the heterogeneity of the data. A smaller spatial unit than a municipality would have been more accurate for the analysis, however, given that most of the cases occurred in rural areas and that correct or full addresses were not given, it would not be possible to classify cases by neighbourhoods or census tracts. This limitation has been partially overcome due to the greatly varied incidence rates reported by the municipalities.

Despite these limitations, the data provided were sufficient to deduce statistically significant information. The use of GIS and spatial analysis tools were important in allowing the authors to attain the objectives proposed.

## Conclusions

We conclude that Acre stands out in the Brazilian national context in view of the high rates of ACL incidence reported in the meso-region of Acre Valley. In addition, the municipalities of Acre had greatly varied incidence rates, ranging from 3.1 to 89.1 cases per 10,000 inhabitant-year. The high rates observed in the micro-region of Brasiléia are related to the occurrence of transmission of the *Leishmania* spp. in the intra/peridomiciliary environment. Further, it seems that the municipality of Sena Madureira is moving towards a similar transmission pattern to that which is occurring in Brasiléia. In the other micro-regions, the pattern of transmission of the disease is related mainly to the forest/sylvatic cycle of ACL.

The municipal/regional surveillance organs should take into account the differences between the transmission patterns of each locality and the identified high-risk cluster in developing actions to mitigate the effects of the zoonosis in the state. Additionally, as the disease is affecting isolated populations, such as chestnut and rubber extractors, farmers, etc., this means health authorities must provide information and campaigns to these populations about the importance of early diagnosis and treatment of ACL, with a view to reducing the appearance of new cases and preventing mucous cases of the disease.

## Additional files

Additional file 1: Multilingual abstracts in the six official working languages of the United Nations. (PDF 682 kb)

Additional file 2: Annual incidence of ACL (cases per 10,000 inhabitant-years) by micro-regions of the state of Acre, Brazil, from 2007 to 2013. (PDF 13 docx)

Additional file 3: Figure: Circles showing locations of detected clusters of ACL cutaneous cases, based on the a) space-time analysis and b) purely spatial analysis, Acre state, 2007 – 2013. (TIF 293 kb)

Additional file 4: Graph: Annual incidence of ACL by micro-regions of Acre, Brazil, 2007 – 2013. (TIF 106 kb)

## Abbreviations

ACL: American cutaneous leishmaniasis
GIS: Geographical information system
IBGE: Brazilian Institute of Geography and Statistics (Instituto Brasileiro de Geografia e Estatística)
RR: Relative risk
