# Supplementary material for: Spatial and temporal distribution of American cutaneous leishmaniasis in Acre state, Brazil
Source: Infect Dis Poverty. 2017 Jun 7;6:99. doi: 10.1186/s40249-017-0311-5 (PMC5461694; doi:10.1186/s40249-017-0311-5)
Supplement: Supplementary file 1 — Multilingual abstracts in the six official working languages of the United Nations. (PDF 682 kb) [file 40249_2017_311_MOESM1_ESM.pdf]

Translation of the abstract into the five official working languages of the United Nations

التوزيع المكاني والزماني لداء الليشمانيا الجلدي الأمريكي في ولاية أكري، البرازيل

ليوناردو أوغوستو كوهارا ملكيور، أندريا فرنانديز بريانتي، فرانسيسكو كيارافالوتي نت

موجز

معلومات أساسية: أفادت ولاية أكري أنها شهدت أكبر عدد من الإصابات بداء الليشمانيا الجلدي الأمريكي (فيما يلي ''الليشمانيا'') في البرازيل في السنوات الأخيرة. وتحاول هذه الدراسة تحديد التجمعات السكانية المعرضة للمخاطر العالية والمنخفضة للإصابة بهذا الداء في المكان والحيز الزمني خلال الفترة من عام 2007 إلى عام 2013 في ولاية أكري، فضلا عن وصف حالات الإصابة به في الزمن وحسب المتغيرات الاجتماعية الديموغرافية.

الأساليب: هذه دراسة إيكولوجية تتألف المجموعة التي خضعت لها من مصابين بالليشمانيا من السكان الأصليين الذين أُبلغ عن وجودهم في بلديات أكري عن طريق نظام مراقبة الأوبئة. واستُخدمت إحصاءات المسح المستخرجة بواسطة برمجيات (SaTScan™) لتحديد مجموعات المكان والحيز الزمني. وبالإضافة إلى ذلك، تم التمييز بين حالات الإصابة حسب الجنس والعمر ومكان الإقامة (في المناطق الريفية أو الحضرية) وحسب توجه الزمن.

النتائج: أَبلغت أكري بمعدل إصابات سنوي قدره 12.4 حالة لكل 10 000 نسمة خلال فترة الدراسة، مع نسب اختلفت إلى حد كبير (انحراف معياري يبلغ 21.8) بين البلديات، وعددها 22 بلدية. وتم الكشف عن تجمع سكني واحد معرض لمخاطر عالية، وثلاثة تجمعات معرضة لمخاطر منخفضة في المكان والحيز الزمني. وظهرت في أربع من المناطق الصغرى الخمس التي تتكون منها أكري توجها زمنيا ثابتا. ويختلف نمط انتقال المرض باختلاف المناطق الصغرى. وعلى العموم، تحدث الإصابة بهذا المرض في أكثر الأحيان بين الشباب الذكور الذين يعيشون في المناطق الريفية.

الاستنتاجات: تحتل أكري مكانة بارزة في السياق الوطني البرازيلي بسبب ارتفاع معدلات الإصابة بالليشمانيا في المنطقة الوسطى من وادي أكري. وترتبط المعدلات المرتفعة المسجلة في منطقة برازيليا الصغرى بحدوث الإصابات بالمرض داخل أماكن الإقامة وفي محيطها، ويبدو أن بلدية سينا مادوريرا تقترب من نمط انتقال مشابه للنمط المسجل في برازيليا. وفي غير ذلك من المناطق الصغرى، يرتبط نمط انتقال المرض أساسا بالدورة تطور الليشمانيا في المناطق المكسوة بالغابات.

Translated from English version into Arabic by asselman, through

TRANSLATORS
WITHOUT BORDERS

美洲皮肤利什曼病在巴西 Acre 州的时空分布

Leonardo Augusto Kohara Melchior, Andréia Fernandes Brilhante, Francisco Chiaravalloti Net

摘要

引言：近年来，巴西 Acre 报告的美国皮肤利什曼病（ACL）发病率最高。本研究旨在从 Acre 州 2007-2013 年期间确定 ACL 在空间和时空上的高低风险聚类，并且根据社会人口学变量描述疾病发生的特征。

方法：这是一项生态学研究，研究人群包括由 Acre 州政府流行病学监测系统所监测到的本地 ACL 病例。SaTScan™软件的扫描统计数据用于识别空间和时空聚类。此外，这些病例的特征包括性别、年龄、家庭情况（农村或城市地区）和时间趋势。

结果：Acre 州在研究期间报告发病率为 12.4 例/万人，其 22 个市县的发病率差异很大（标准差为 21.8）。在空间和时空上发现了 1 个高风险聚类和 3 个低风险聚类。Acre 州的 5 个“微区”中有 4 个呈现出稳定的时间趋势，传播趋势根据“微区”区域而变化。一般来说，该病在年轻人、男性和生活在农村的人群中发病率较高。
结论：Acre Valley 中部地区的 ACL 发病率很高，因此在在巴西 Acre 州受到关注。Brasiléia “微区”的高发病率与疾病的住所内和住所周围的发生有关，而且 Sena Madureira 市似乎正在接近类似于 Brasiléia 的传播模式。在其他“微区”，疾病传播情况主要与 ACL 的森林栖息周期有关。

Translated from English version into Chinese by Xin-Yu Feng, edited by Pin Yang

CHINESE CENTER FOR DISEASE CONTROL AND PREVENTION
NATIONAL INSTITUTE OF PARASITIC DISEASES

Distribution spatiale et temporelle de la leishmaniose cutanée américaine dans l’état d’Acre au Brésil

Leonardo Augusto Kohara Melchior, Andréia Fernandes Brilhante, Francisco Chiaravalloti Ne

RÉSUMÉ

Contexte : Ces dernières années, c’est l’état d’Acre qui a rapporté la plus forte incidence de leishmaniose cutanée américaine au Brésil. La présente étude a pour but d’identifier les agrégations spatiales et spatiotemporelles à haut et bas risque de la leishmaniose pendant la période de 2007 à 2013 dans cet état et de caractériser l’incidence de la maladie dans le temps et en fonction de variables sociodémographiques.
Méthodes : Cette étude est une étude écologique dont la population est constituée par des cas autochtones de leishmaniose cutanée américaine déclarés dans les municipalités de l’état d’Acre par un système de surveillance épidémiologique. Les statistiques d’analyse du logiciel SaTScan™ ont été utilisées pour identifier les agrégats spatiaux et spatiotemporels. Les cas ont en outre été caractérisés par sexe, âge, lieu d’habitation (zone rurale ou urbaine) et tendance temporelle.
Résultats : Acre a déclaré une incidence de 12,4 cas par 10 000 années-habitant sur la période de l’étude, avec des taux très variable entre ses 22 municipalités (écart-type de 21,8). Un agrégat à haut risque et trois à faible risque ont été détectés dans l’espace et dans l’espace-temps. Quatre des cinq microrégions d’Acre présentaient une tendance temporelle stationnaire. Le profil de transmission était variable selon les microrégions. Dans l’ensemble, la maladie était plus fréquente chez les sujets jeunes, ceux de sexe masculin et ceux vivant dans les zones rurales.
Conclusions : Dans le contexte national brésilien, Acre s’est fait remarquer par la forte incidence de la leishmaniose cutanée américaine dans la mésorégion de la Vallée de l’Acre. Le taux élevé de leishmaniose cutanée dans la microrégion de Brasiléia est lié à la présence intra- et péridomiciliaire de la maladie, et il semble que la municipalité de Sena Madureira se rapproche du même schéma de transmission. Dans les autres microrégions, le profil de transmission coïncide, pour l’essentiel, avec le cycle forestier/sylvatique de la leishmaniose cutanée américaine.

Translated from English version into French by Suzanne Assenat, through

Территориально-временное распространение американского кожного лейшманиоза в бразильском штате Акри

Леонарду Аугусту Кохара Мелшиор, Андрэйа Фернандес Брильянти, Франсиску Шиаравалоти Нет

АННОТАЦИЯ

Актуальность исследования: Согласно данным за последние годы, в штате Акри отмечен самый высокий уровень заболеваемости американским кожным лейшманиозом (АКЛ) в Бразилии. Настоящее исследование ставит целью выявить через территориальный и территориально-временной анализ зоны повышенного и сниженного риска заболеваемости АКЛ с 2007 по 2013 гг. в штате Акри, а также охарактеризовать динамику возникновения заболевания в соответствии с социально-демографическими переменными.

Методы исследования: Данное исследование является экологическим, предметом исследования стало автохтонное население, среди которого система надзора за эпидемиологическими поражениями муниципалитета Акри зафиксировала случаи заболеваемости АКЛ. Для определения территориальных и территориально-временных групп анализа был использован статистический метод сканирования в программе SaTScan™. Кроме того, все случаи были проанализированы с точки зрения пола, возраста, места проживания (в сельской или городской местности) и наличия тенденции к временному распространению.

Результаты исследования: В исследуемый период уровень заболеваемости в Акри составил 12,4 случая на 10,000 жителей в год, при этом полученные результаты распределены во многом неравномерно по 22 муниципалитетам (стандартное отклонение составило 21,8). В результате территориального и территориально-временного анализа были обнаружены одна зона повышенного риска и три зоны сниженного риска. В четырех из пяти микрорайонов Акри была выявлена постоянная тенденция к временному распространению. Показатели передачи заболевания различались в зависимости от микрорайона. В целом, болезнь значительно чаще поражала молодых людей мужского пола, проживающих в сельских районах.

Заключение: В национальном контексте Бразилии штат Акри выделился высокими уровнями заболеваемости АКЛ в центральном регионе долины Акри. Высокие уровни заболеваемости в этом микрорайоне Бразилеи связаны с внешним (внутренним) местом возникновения заболевания. Предположительно, в муниципалитете Сена-Мадурейра будет действовать механизм передачи заболевания, схожий с путем передачи этой болезни в Бразилее. В других микрорайонах показатели, относящиеся к передаче заболевания, в основном связаны с лесным (природным) или сильватическим циклом АКЛ.

Translated from English version into Russian by Ekaterina Verma, through

TRANSLATORS
WITHOUT BORDERS

Distribución espaciotemporal de la Leishmaniasis cutánea americana en el estado brasileño de Acre

Leonardo Augusto Kohara Melchior, Andréia Fernandes Brilhante, Francisco Chiaravalloti Net

RESUMEN

Introducción: El estado brasileño de Acre cuenta con la mayor incidencia de Leishmaniasis cutánea americana (LCA) de los últimos años en todo el país. Este informe tiene como misión la identificación de las aglomeraciones de alto y bajo riesgo de LCA tanto a nivel espacial como a nivel espaciotemporal entre los años 2007 y 2013 en Acre y también se ocupa de clasificar la ocurrencia de la enfermedad en un período determinado y de acuerdo a variables sociodemográficas.

Metodología: El presente documento es un estudio ecológico cuya población de estudio son los casos autóctonos de LCA que las distintas municipalidades de Acre han notificado mediante un sistema de vigilancia epidemiológico. Se han empleado estadísticas escaneadas del programa SaTScan™ para identificar grupos espaciales y espaciotemporales. Los casos de estudio también se han clasificado por edad, sexo, situación habitacional (zonas rurales o urbanas) y tendencias temporales.

Resultados: El estado de Acre informó de la existencia de un nivel de incidencia anual de 12,4 casos por cada 10,000 habitantes durante el estudio. Estos niveles fluctuaban enormemente (con una desviación típica de 21,8) entre las 22 municipalidades. Se detectó una aglomeración de alto riesgo y tres de bajo riesgo tanto a nivel temporal como espacio temporal. Cuatro de las cinco micro regiones de Acre presentaban una tendencia temporal estacionaria. El perfil de transmisión variaba de acuerdo a cada microregión. En general, la enfermedad ocurría más a menudo entre jóvenes, hombres y habitantes de zonas rurales.

Conclusiones: Acre ha resaltado en el contexto nacional brasileño debido a sus altos niveles de incidencia de LCA en la región central del Valle de Acre. Estos altos niveles de incidencia en la microregión de Brasiléia están relacionados con la ocurrencia de la enfermedad dentro y alrededor del domicilio y se puede observar cómo la municipalidad de Sena Madureira se acerca al modelo de trasmisión similar al de Brasiléia. En otras microrregiones, el perfil de la trasmisión de la enfermedad está principalmente relacionado al ciclo selvático de LCA.

Translated from English version into Spanish by Mar PelaezMunoz, through

TRANSLATORS
WITHOUT BORDERS
