# Supplementary material for: Spatial and temporal distribution of American cutaneous leishmaniasis in Acre state, Brazil
Source: Infect Dis Poverty. 2017 Jun 7;6:99. doi: 10.1186/s40249-017-0311-5 (PMC5461694; doi:10.1186/s40249-017-0311-5)
Supplement: Supplementary file 2 — Annual incidence of ACL (cases per 10,000 inhabitant-years) by micro-regions of the state of Acre, Brazil, from 2007 to 2013. (PDF 13 docx) [file 40249_2017_311_MOESM2_ESM.docx]

|  | 2007 | 2008 | 2009 | 2010 | 2011 | 2012 | 2013 |
| --- | --- | --- | --- | --- | --- | --- | --- |
| *State* |  |  |  |  |  |  |  |
| Acre | 13.0 | 14.2 | 12.4 | 12.7 | 10.9 | 13.8 | 10.3 |
| *Meso-region* |  |  |  |  |  |  |  |
| Vale do Acre | 14.2 | 16.0 | 14.1 | 14.5 | 11.3 | 14.4 | 11.2 |
| Vale do Juruá | 9.9 | 9.8 | 8.3 | 8.1 | 9.9 | 12.2 | 7.9 |
| *Micro-region* |  |  |  |  |  |  |  |
| Brasiléia | 46.0 | 47.6 | 46.2 | 52.0 | 40.3 | 47.6 | 39.7 |
| S. Madureira | 23.8 | 32.9 | 24.8 | 29.3 | 24.8 | 38.6 | 32.4 |
| Tarauacá | 15.5 | 15.0 | 13.3 | 13.0 | 13.3 | 16.4 | 6.4 |
| Cruz. do Sul | 6.7 | 6.9 | 5.5 | 5.3 | 8.0 | 9.9 | 8.7 |
| Rio Branco | 8.5 | 9.3 | 8.2 | 7.4 | 5.6 | 6.7 | 4.6 |

Table - Annual incidence of ACL (cases per 10,000 inhabitant-years) by micro-regions of the state of Acre, Brazil, from 2007 to 2013.
