# Supplementary figures and images for: Spatial and temporal distribution of American cutaneous leishmaniasis in Acre state, Brazil
Source: Infect Dis Poverty. 2017 Jun 7;6:99. doi: 10.1186/s40249-017-0311-5 (PMC5461694; doi:10.1186/s40249-017-0311-5)

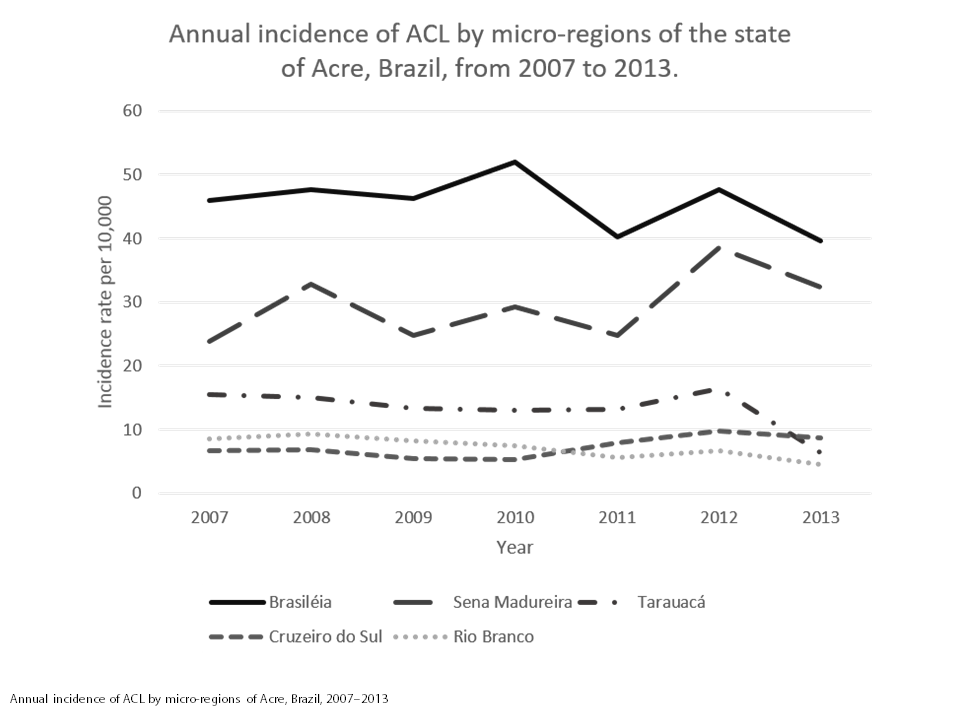

Supplement: Supplementary file 3 — Figure: Circles showing locations of detected clusters of ACL cutaneous cases, based on the a) space-time analysis and b) purely spatial analysis, Acre state, 2007 – 2013. (TIF 293 kb) [file 40249_2017_311_MOESM3_ESM.tif]
